# Incident Angle Sensing and Adaptive Control of Scattering by Intelligent Metasurface

**DOI:** 10.1002/advs.202406841

**Published:** 2024-08-29

**Authors:** Yong Han Liu, Xu Hang Li, Ze Tong Zhang, Kai Nan Qi, Lan Lu, Shi Yu Wang, Yun Bo Li

**Affiliations:** ^1^ State Key Laboratory of Millimeter Waves Southeast University Nanjing 210096 China; ^2^ Science and Technology on Electromagnetic Scattering Laboratory Beijing 100854 China

**Keywords:** adaptive control of scattering wave, incident angle sensing, intelligent metasurface, low scattering, retroreflector

## Abstract

The passive sensing and active control of electromagnetic (EM) waves have always been attractive in electronic and information areas, especially during the intelligent era. Here a new method is presented to achieve the angle sensing of incident wave and adaptive control of backward scattering using the intelligent metasurface. The proposed unit cells have the ability to dynamically manipulate the receiving and reflection of the EM energy respectively. The angle sensing of incident waves can be actualized using the method of compressive sensing based on multiple receiving patterns, which are generated by randomly switching the receiving and reflection states of the unit cells. Afterward, the customized performances of backward scattering waves according to the cognitive incident angle can be realized by controlling the programmable reflective phases of unit cells correspondingly. One prototype composed of the metasurface and the module for sensing and adaptive feedback control is fabricated. The whole intelligent metasurface with customizing the function of retro‐reflection or low scattering is measured without human intervention and the good results acquired can verify the validity of the proposed concept and design.

## Graphical abstract

An intelligent metasurface with the abilities of electromagnetic receiving and reflection through co‐aperture design is proposed to achieve the angle sensing of incident waves and adaptive control of backward scattering without human intervention. According to the cognitive incident angle, the backward scattering waves can be controlled and customized. The measurement results verify the validity of the proposed concept and design.

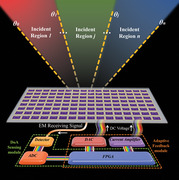

## 1 Introduction

Intelligent metasurface,^[^ ^1^, ^2^, ^3^ ^]^ achieving the EM sensing and feedback control without human intervention, can be regarded as the EM platform or system which is composed of the programmable metasurface array, baseband circuit, and reconstruction algorithms. Compared with traditional passive metasurfaces,^[^ ^4^, ^5^, ^6^, ^7^, ^8^, ^9^, ^10^, ^11^, ^12^, ^13^ ^]^ intelligent metasurfaces exhibit three critical performances: the ability of EM environment detection, adaptive control according to customized functions, and EM feature configurability. Predictably, the introduction of intelligent concepts and technology will make the metasurface much smarter and more flexible.

In order to realize the concept and design of intelligent metasurfaces, the technologies of EM active control^[^ ^14^, ^15^, ^16^, ^17^, ^18^ ^]^ are essential, including the methods of electrical control, optical control, microfluidic control, mechanical control, and so on. Integrating active components such as PIN diodes,^[^ ^19^, ^20^, ^21^, ^22^ ^]^ varactors,^[^ ^23^, ^24^, ^25^, ^26^, ^27^ ^]^ graphene,^[^ ^28^, ^29^ ^]^ liquid crystals,^[^ ^30^ ^]^ or power amplifiers (PAs)^[^ ^31^, ^32^ ^]^ onto the meta‐atoms enables the real‐time electrical control of the whole metasurface. The holograms can be dynamically generated by a programmable reflective phase with switching PIN diodes on each meta‐atom.^[^ ^20^ ^]^ The metallic objects illuminated by diverse apertures actualized by electrically programmable metasurface can be reconstructed with the inverse method.^[^ ^21^ ^]^ The adaptive beamforming and power allocation of the spatial EM beams are implemented separately using the programmable metasurface, in which the unit cells can actualize the independent control of the transmitting amplitude and phase.^[^ ^26^ ^]^ The EM features of spatial non‐reciprocity can be realized by active metasurfaces with integrating PA devices^[^ ^31^, ^32^ ^]^ which have the performance of unidirectional transmission. The transmitting polarization can be reconfigurable by changing the coupling conditions between the plasmonic modes and the binary isomeric states of an ethyl red switching layer through light stimulation.^[^ ^33^ ^]^ Moreover, by injecting solutions with a different refractive index into the polymeric microfluidic channel, the narrow band reflection peak, and the corresponding distinct colors of a TiO_2_ metasurface can be precisely controlled.^[^ ^34^ ^]^ Mechanical control involves operations such as stretching, and compressing to manipulate the deformation of structures on the units of metasurfaces, thereby exhibiting remarkable resonance shifts.^[^ ^35^ ^]^ Different working modes can be controlled to modulate the reflection beam spin‐selectively by folding and stretching the metasurface.^[^ ^36^ ^]^ Recently, the dynamic control of spatial radiation and reflection has been actualized by co‐aperture active metasurface with manual switching under the same frequency band and polarization.^[^ ^37^, ^38^ ^]^

The information sensing of incident waves mainly including angle, frequency, and polarization is also an important component in the design of intelligent metasurfaces. The direction of arrival (DoA) estimation was achieved by multiple radio frequency (RF) channels in the traditional phased array by applying the post‐processing algorithms of the multiple signal classification (MUSIC) algorithms^[^ ^39^ ^]^ and the estimating signal parameters via the rotational invariance techniques (ESPRITs) algorithm.^[^ ^40^, ^41^ ^]^ To address the drawbacks of high costs associated with multiple sensors in a phased array, the information acquirement of DoA can be realized by space‐time coding (STC) metasurfaces^[^ ^42^, ^43^, ^44^ ^]^ with only one sensing channel. Applying diverse apertures generated by metasurface composed of massive randomly switchable unit cells, the issue of DoA estimating can be solved with the method of compressive sensing.^[^ ^45^ ^]^ For the STC method, the solving time of DoA estimation is less with a low resolution of spatial angle; and for the diverse apertures‐based method, the solving time of DoA estimation is larger with a higher resolution of spatial angle. The EM frequency can also be detected based on an algorithm, consisting of multiple applications of lowpass filtering and decimation, frequency estimation by linear prediction, and digital heterodyning.^[^ ^46^ ^]^ In addition, the feature of EM polarization can be recognized by the sensors composed of measured six vector components using the ESPRIT‐based algorithm.^[^ ^47^ ^]^

Recently, the development of intelligent metasurfaces, which combine active control techniques with passive sensing technologies, has emerged as a significant scientific research hotspot. By detecting the rotating states of the metasurface itself by integrating the commercial three‐axis gyroscope, the reflective EM beam can be adaptively manipulated to the required scattering directions.^[^ ^48^ ^]^ Using a commercial direction finder, an angular‐adaptive reconfigurable spin‐locked metasurface retroreflector can be controlled dynamically and continuously by altering its orientation states by individually addressing each mechanically rotational meta‐atom.^[^ ^49^ ^]^ By applying the external eight dipole antennas outside of the metasurface for sensing the information of incident angle, frequency, and polarization of coming wave, the scattering beams can be correspondingly operated by the proposed neuro‐metasurface with the help of the deep learning method.^[^ ^50^ ^]^ The adaptive frequency manipulation can be implemented through a cognitive metasurface comprising patterned graphene sandwich structures and an external RF detector beside the metasurface to achieve adaptive EM absorption.^[^ ^51^ ^]^ Combining the RF power received by the meta‐atom in the center of the metasurface with the baseband frequency‐recognition module, the frequency of a coming wave can be determined and the customized diverse EM scattering functions corresponding to frequencies can be adaptively achieved with the independent control of the reflective phase and amplitude.^[^ ^52^ ^]^ With THz wave detection and modulation capabilities, an integrated self‐adaptive metasurface (SAM) is designed based on the phase change material to deflect THz beams over an angle range.^[^ ^53^ ^]^ However, the EM sensing and feedback control under the same aperture of the whole metasurface has not been proposed.

In this paper, we propose and experimentally demonstrate an intelligent co‐aperture multiplexing metasurface that can realize the spatial wave receiving to sense the angle information of incident wave and adaptively manipulating the backward scattering wave, including the customized function of retro‐reflection or low scattering without human intervention. A homemade intelligent baseband module for recognition and feedback control is located at the back of the metasurface. The experimental results indicate that the proposed design possesses the ability of DoA estimation and the good achievement of adaptive control for backward scattering waves.

## 2 Results and Discussion

**Figure 1:**
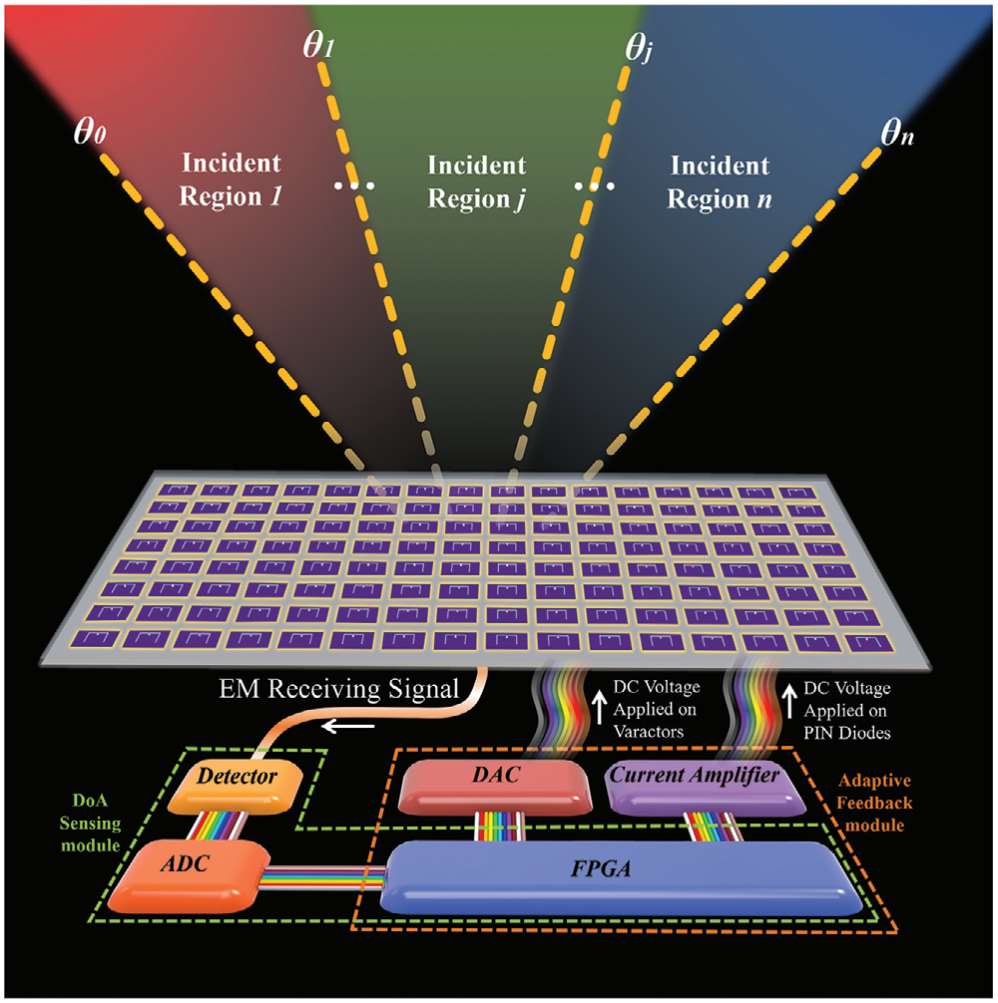
The schematic diagram of the proposed intelligent metasurface can realize the angle sensing of coming waves and feedback control of backward scattering waves.

The schematic diagram and design method of the proposed work are shown in **Figure** **1**. The intelligent metasurface can have both EM receiving and reflection abilities realized by only one multiplexing co‐aperture without external devices for sensing. For the whole flow path of the intelligent metasurface, the angle information of the coming wave should be acquired first by compressive sensing method for processing the received multiple EM signals using random diverse receiving apertures generated by dynamic control. Afterward, the performances of backward scattering waves depended on the customized EM functions corresponding to the acquired angle information can be adaptively manipulated without human intervention. Both the sensing and feedback operation can be realized in one homemade baseband module shown in the lower part of Figure 1.

In our work, the customized functions of backward scattering waves are determined as retro‐reflection and low scattering, which are very common in the field of EM object characteristics. We have separated the space into several regions based on angle domain, and each region can be freely defined as the function of retro‐reflection or low scattering depending on the pre‐stored mapping relationship between the acquired angle information and customized scattering functions in the device of field‐programmable gate array (FPGA) included in the intelligent module located on the back of the metasurface.

### 2.1 The Design of the Unit Cell for the Proposed Intelligent Metasurface

**Figure 2:**
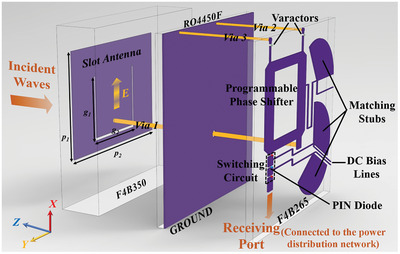
The exploded view for the unit cell of the proposed metasurface for achieving the control of EM receiving or scattering. The period of the unit cell is 25 mm. The main parameters of the top slot antenna are *p*_1_ = 15.05, *p*_2_ = 20.07, *g*_1_ = 9.25, *g*_2_ = 9.94 mm.

To accomplish the design of intelligent metasurface illustrated above, the unit cell of the metasurface should have the EM switchable abilities of receiving and scattering. In addition, the EM features of receiving and scattering can be dynamically controlled in real‐time. Accordingly, the proposed active unit cell with a period of 25 mm is shown in **Figure** **2**, and it mainly consists of a slot antenna with linear polarization, a programmable phase shifter, and a switching circuit. The slot antenna placed on the top of the whole unit cell, can receive the coming EM signals, or re‐radiate into space and the U‐shaped slot antenna is preferred due to the consideration of working bandwidth. For the bottom layer as illustrated in Figure S1 of Section S1 (Supporting Information), the programmable phase shifter is applied to realize the continuously dynamic control of the reflective phase, and the switching circuit mainly composed of the PIN diode is introduced to select the states of EM receiving or reflection (scattering) of the unit cell.

Thus, the EM power flow captured by the top slot antenna can be decided whether to be received or not into the sensing module through the power distribution network. For further DoA sensing work, only the variation of receiving amplitudes is required without changing the states of the programmable phase shifter. For further scattering beam control, the closed PIN diode can make the unit cell to be a reflective state. Thus, the programmable phase shifter can be passed through twice by propagating waves inside the unit cell and only two varactors are needed to achieve the 360 degree reflective phase coverage accordingly. The topological of the EM power flow within the unit cell is depicted in Figure S2 of Section S1 (Supporting Information).

Due to the massive integrated active components and complex EM power flow in the proposed unit cell, the U‐shaped slot antenna is selected with optimized tailoring to guarantee the working bandwidth. The determined sizes of the slot antenna are given in Figure 2, and it is located on the substrate of F4BTM350 which has a dielectric constant of 3.5 and a dielectric loss tangent of 0.0025 with a thickness of 4 mm. The programmable phase shifter and the switching circuit are both located on the substrate of F4BTM265 characterized by a dielectric constant of 2.65 and a dielectric loss tangent of 0.0015 with a thickness of 0.5 mm. The bonding film material chosen as Rogers 4450F with a thickness of 0.1 mm is employed to combine the top and bottom layers. The dielectric constant and dielectric loss tangent of the bonding film are 3.52 and 0.009 respectively.

**Figure 3:**
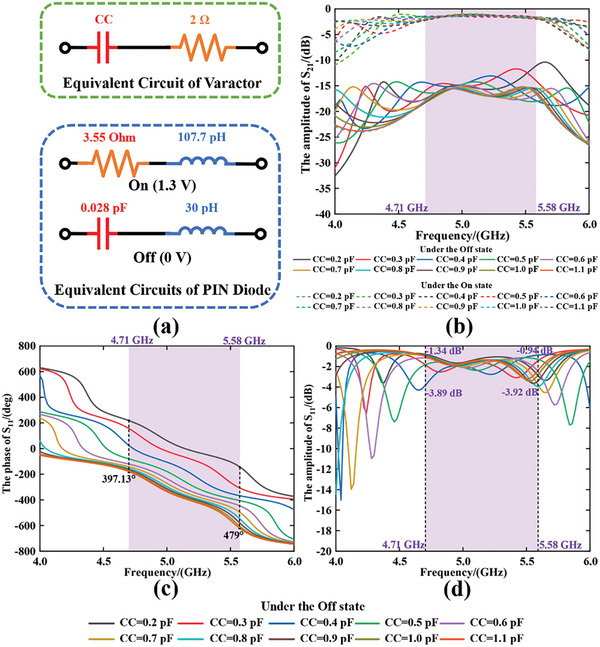
a) The equivalent circuits of the varactor (CC varies from 0.2 to 1.1 pF) and the PIN diode in design. The simulation results of b) the amplitude of S_21_ when the CC of varactor changes from 0.2 to 1.1 pF under both the On and Off states of the PIN diode; c) The phase of S_11_ and d) the amplitude of S_11_ while CC changes from 0.2 to 1.1 pF.

To further achieve dynamic EM functions of scattering control, the programmable phase shifter located at the bottom layer of a unit cell is the core design. A reflective phase coverage larger than 270 degrees with lower fluctuation of reflective amplitude is required in the design of a programmable phase shifter. The selected reflection‐type programmable phase shifter is mainly composed of two parts: the two Π‐type circuits (including two varactors) and the 3dB branch‐line coupler respectively. The type of MACOM MA46H120 chosen as the varactor with the equivalent circuit shown in **Figure** **3**a is composed of a capacitor (with the capacitance ranging from 0.2 to 1.1 pF) and a 2 Ω resistor in series. By continuously varying the DC voltage on the varactors through DC bias lines, the phase shift can be dynamically manipulated accordingly. The type of MACOM MADP‐000907‐14020P is selected as PIN diode whose equivalent circuit under the On and Off states are presented in Figure 3a respectively. All the equivalent circuits are extracted based on the *SnP* files provided by the manufacturer. For the active design of the programmable phase shifter and switching circuit, the matching stubs connected with the bias line are introduced to prevent the power of RF signals into the DC control module. Additionally, one capacitor of 1nF is added to separate the parts of the programmable phase shifter and switching circuit to make them be controlled independently.

The proposed unit cell is analyzed under full‐wave method by the commercial software of High Frequency Structure Simulator (*HFSS*). The periodic boundary conditions are set around the whole unit cell. The Floquet port defined as port_1 is set at the top surface of the air box in *HFSS* to be selected as an incident port. The lumped port defined between the microstrip line and ground in simulation is set as port_2 to be considered as the receiving port. Moreover, the equivalent circuits of the PIN diode and varactor are loaded into the unit cell by using the RLC boundaries to mimic the open or closed state of PIN diode and various capacitances of the varactor correspondingly.

Accordingly, the simulated amplitude of S_21_ while varying the capacitance values of the varactor under the On and Off states of the PIN diode is depicted in Figure 3b, exhibiting a distinct amplitude difference under the frequency band of 4.71 to 5.58 GHz. The average amplitude difference within the frequency band is ≈14.1 dB, and further DoA estimation can benefit from the larger difference of the switchable receiving amplitudes which can lead to a larger difference of the random receiving patterns of metasurface correspondingly. And for the reflective state of the unit cell when the PIN diode is Off, the simulation results of phase and amplitude of S_11_ are given in Figure 3c,d respectively with changing the capacitance values of the varactor from 0.2 to 1.1 pF. The reflective phase can be continuously manipulated within 360° with variations of reflective amplitude less than 3 dB in the operating frequency band, and a minimum variation of 0.4 dB is observed under the 4.9 GHz due to the simulation results.

### 2.2 Implementation of Intelligent Metasurface

Based on good simulation results of the unit cell, the sample fabrication of the metasurface is considered in sequence. Due to the complex design composed of massive active components, the waveguide measurement (see Section S2, Supporting Information) is strongly required for initially verifying the EM features of a proposed unit cell. Because the obtained results of waveguide measurement are similar to our simulation ones, the whole metasurface is encouraged to be fabricated and be moved to accomplish the entire calibration of electrically controlled EM performances. In detail, the fabricated prototype of metasurface using PCB (Printed Circuit Board) technology is composed of 8 × 16 unit cells total. Copper is selected as the metal component of the sample, and it is coated with tin to prevent oxidation.

Due to the requirements of EM functions achieved by the proposed intelligent metasurface, the performances of EM receiving, and reflection should be preliminarily verified based on the calibration of the whole metasurface. In detail, the SMA connector loaded on the terminal receiving port of the metasurface and one horn antenna located in the far field are connected to Port_2 and Port_1 of the Vector Network Analyzer (VNA, Agilent N9928A) respectively. The bias voltages loaded on the varactors and PIN diodes are supplied by the DAC module (NI PXIe‐6739) under 16‐bit control, which can be regarded as continuous manipulation.

**Figure 4:**
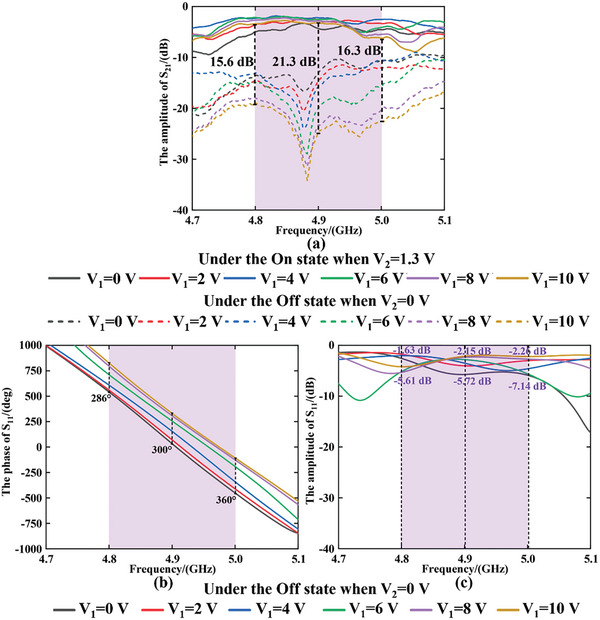
The measurement results of a) the amplitude of S_21_ when the voltage of varactors (V_1_) changes from 0 to 10 V under On and Off states of the PIN diodes (V_2_); b) The reflective phase (the phase of S_11_) and c) the reflective amplitude (the amplitude of S_11_) while the voltage of varactors (V_1_) changing from 0 to 10 V under the Off state of the PIN diodes.

By maintaining all unit cells under the same programmable control, the measured amplitude and phase results of the transmission coefficient and reflection coefficient are depicted in **Figure** **4**, respectively. The fabricated metasurface operates within a frequency band ranging from 4.8 to 5.0 GHz, centered at a frequency of 4.9 GHz, with a relative bandwidth of 4.08%. When V_1_ (the voltage loaded on the varactors) is preserved at a fixed value (10 V in our measurement), the receiving amplitude (S_21_) difference above 15 dB shown in Figure 4a is accomplished by switching V_2_ between 0 and 1.3 V. When V_2_ maintained at 0 V (the Off state of PIN diode) corresponding to the reflective state of metasurface, the reflective phase and amplitude of S_11_ are obtained, as depicted in Figure 4b,c. Considering the performances of the reflective phase and amplitude jointly, the center working frequency of 4.9 GHz is preferred under which the phase has the ability of 300‐degree coverage while the fluctuation of amplitude does not exceed 4 dB during the phase variation. The mapping relationship between the measured reflective phase and DC voltage (V_1_) under 4.9 GHz can be obtained by the method of polynomial fitting (see Section S3, Supporting Information), and the further control of scattering beams can be realized based on the synthesized method in far‐field accordingly.

### 2.3 The Sensing of Incident Angle and Adaptive Control of Scattering by Intelligent Metasurface

**Figure 5:**
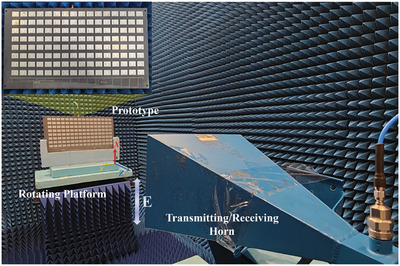
The experiment setup of far‐field measurement in a microwave anechoic chamber.

Based on the good performances of EM receiving and reflection shown in Figure 4, the measurement for sensing and adaptive feedback control using the intelligent metasurface can be launched. The intelligent metasurface placed at the 2D rotating platform in the microwave anechoic chamber shown in **Figure** **5** is illuminated by the horn antenna located in the far field under vertical polarization. For the proof of the concept, the measurement is only conducted in the azimuth plane and the column control (see Section S4, Supporting Information) of the intelligent metasurface is only needed with consideration of low complexity and low cost.

Due to the main angle‐dependent EM functions to be achieved, the angle between the incident wave from the horn antenna and the normal direction of the metasurface under the current state of rotation should be recognized first. It can be solved by compressing method based on diverse receiving apertures generated by the metasurface by randomly switching the receiving states of On and Off in each unit cell. The large difference between the amplitude results of S_21_ under the two states shown in Figure 4a, can ensure the large discrimination of the receiving apertures. Accordingly, the 60 different receiving patterns in the far field corresponding to 60 various apertures of metasurface are collected with the platform rotating by each azimuth angle under the frequencies from 4.8 to 5.0 GHz (see Section S5, Supporting Information).

**Figure 6:**
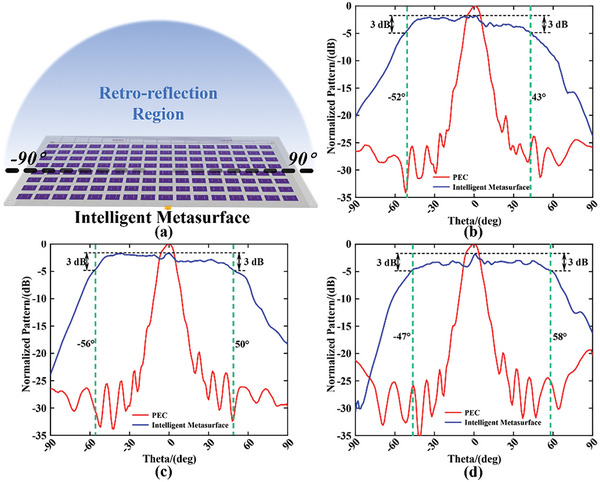
The measurement of case I includes one sub‐region. a) The schematic of the function definition in case I is achieved by intelligent metasurface. The measurement backward scattering patterns normalized to the results of PEC under frequencies of b) 4.8, c) 4.9, and d) 5.0 GHz.

**Figure 7:**
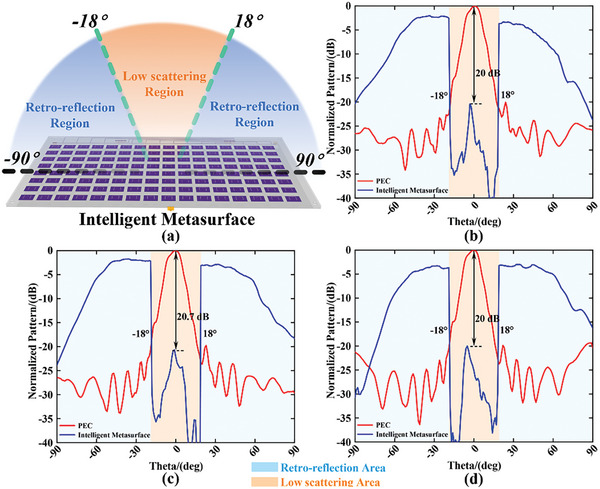
The measurement of case II includes three sub‐regions. a) The schematic of the function definition in case II achieved by intelligent metasurface. The measurement backward scattering patterns normalized to the results of PEC under frequencies of b) 4.8, c) 4.9, and d) 5.0 GHz.

**Figure 8:**
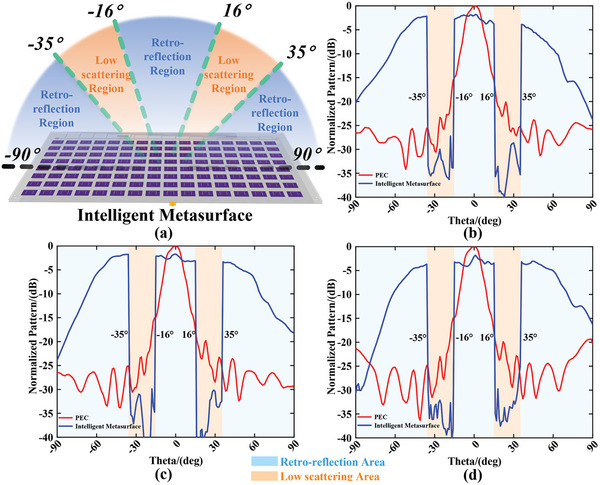
The measurement of case III includes three sub‐regions. a) The schematic of the function definition in case III achieved by intelligent metasurface. The measurement backward scattering patterns normalized to the results of PEC under frequencies of b) 4.8, c) 4.9, and d) 5.0 GHz.

The angle information of incident wave can be estimated by mathematical calculations discussed in the Experimental methods section, and the sensing results of DoA with high accuracy (see Section S6, Supporting Information) are actualized in our measurement using DoA sensing section of our homemade intelligent module (see Section S7, Supporting Information). The adaptive control of the backward scattering wave can be guided by the sensing information of DoA due to the pre‐stored mapping relationship in the register of FPGA section in an intelligent module. The EM functions of retro‐reflection and backward low‐scattering (implemented by the phase cancellation of metasurface) are preferred to be verified in measurement, and their theoretical implementation is discussed in the Experimental Section. The above functions can be assigned into customized separated regions in the angle domain arbitrarily. Three selected cases with one, three, and five spatial sub‐regions are launched with the angles ranging from −90° to +90° separated as 1° intervals. The scattering field is measured by S_11_ parameter in VNA which is loaded the gate in the time domain for decreasing experimental noise. The discrete data will be sampled by VNA under each rotation of 2D rotating platform at intervals of 1°. The performances of retro‐reflection and low‐scattering are observed clearly in **Figures** **6**, **7**, and **8** respectively.

Due to the measurement results shown in Figure 6, the reduction of ≈1.8 dB compared with the Perfect Electric Conductor (PEC) in the broadside is observed. The spatial angle ranges for beam retro‐reflection within the 3dB reduction are 95°, 106°, and 105° corresponding to the measurement frequencies of 4.8, 4.9, and 5.0 GHz.

Due to the measurement results shown in Figures 7 and 8, both functions of retro‐reflection and backward low‐scattering assigned to different sub‐regions are achieved. The corresponding sharp decline of the backward scattering intensity can be observed between regions of retro‐reflection and low‐scattering due to the customized EM functions pre‐stored in our intelligent module located at the backside of the metasurface. For the performances of low scattering, more than 20 and 25 dB differences compared with PEC scattering in broadside within the spatial angles we defined are observed under the frequencies of 4.8, 4.9, and 5.0 GHz in Figure 7 and 8 respectively. All of the measurement results are obtained by angle sensing with the resolution of 1°, and the time for jointly sensing and feedback controlling in the total homemade intelligent module without human intervention should be ≈1.459 ms.

For the difference between the simulation results of the unit cell and the experimental calibration results of the whole metasurface, the reasons can be summarized by the factors below: 1) by loading and welding of massive positive devices on the prototype of the metasurface, some error will be introduced inevitably; 2) many DC bias lines around the metasurface without shielding form noneffective operational section of the metasurface which may lead interference of the measurement results; 3) the unit cells located at the edge of the metasurface do not satisfy periodic boundary conditions which is different from the ideal periodic boundaries in simulation. As for the imperfect measurement results of scattering waves, the reasons can be mainly attributed to the mutual interference between active unit cells with different programmable coding, the inaccuracy of DC voltage loaded on varactors, and manmade errors of sample fabrication and experiment.

For the working distance of the whole intelligent metasurface, the sensing of DoA is the key point. Additional low noise amplifiers (LNAs) are required in the receiver to detect the weaker incident signals to realize the DoA estimation under a longer working distance. For the scattering control, the better results of backward reflection will be realized under longer distances due to the better performance of the quasi‐plane wave.

The concept and design of the proposed intelligent metasurface can be considered as one new method to realize the control of the backward scattering waves. Compared with the classical designs of the Luneburg lens,^[^ ^54^ ^]^ corner reflectors,^[^ ^55^ ^]^ array with circuit of conjugate phase,^[^ ^56^ ^]^ and array of Van Atta^[^ ^57^ ^]^ which have different advantages or disadvantages, the backward scattering functions can be customized arbitrarily in the angle domain by intelligent metasurface. Although the response of sensing and feedback control is time‐consuming, the design of an intelligent metasurface will benefit from the development of a digital circuit for fast signal processing.

## 3 Conclusion

In this paper, a new method for joint angle sensing of incident waves and feedback control of backward scattering waves is proposed by using an intelligent metasurface composed of EM programmable structures and a homemade intelligent module. The programmable metasurface integrating massive active devices, it has the ability to dynamically manipulate the EM receiving and scattering using the multiplexing aperture method. And for the intelligent module, can realize the sensing and adaptive feedback control under incorporated design by utilizing the technology of baseband circuits mainly including the FPGA. The metasurface‐based intelligent system has been fabricated and measured, and the EM functions of retro‐reflection and low scattering assigned customized into separate sub‐regions in the angle domain have been actualized by acquiring the information of DoA first. Due to the good performances shown in our measurement results under the frequencies from 4.8 to 5.0 GHz, the proposed new method for achieving the customized and adaptive control of backward scattering waves by intelligent metasurface has been verified. The proposed intelligent metasurface can be applied to adaptive control of scattering wave depending on arbitrarily customized angle regions, some of which are defined as the function of retro‐reflection like the passive “lighthouse” for partners, and others are defined as low‐scattering to be low detectable for non‐partners.

## 4 Experimental Section

### The Sensing Method for the Estimation of DoA

Compressive sensing was chosen as the method for DoA estimation in this work. Similar to the computational imaging methodologies,^[^ ^58^, ^59^ ^]^ the proposed metasurface was utilized to randomly generate far‐field patterns *F*(ϕ) for receiving the incident waves coming from various angles to construct the sensing matrix *G* described as:

(1) $$G=\left(\begin{array}{cccc} F_{1}\left(\phi_{1}\right) & F_{1}\left(\phi_{2}\right) & \cdots & F_{1}\left(\phi_{M}\right)\\ F_{2}\left(\phi_{1}\right) & F_{2}\left(\phi_{2}\right) & \cdots & F_{2}\left(\phi_{M}\right)\\ \vdots & \vdots & \ddots & \vdots \\ F_{L}\left(\phi_{1}\right) & F_{L}\left(\phi_{2}\right) & \cdots & F_{L}\left(\phi_{M}\right) \end{array}\right)$$

where *L* represents the number of random receiving patterns. If the whole space in the angle domain was divided into *M* equivalent angles at the azimuth plane, the dimension of the sensing matrix *G* can be determined as *L* × *M*. When the intelligent metasurface with various receiving patterns was illuminated by the incident wave under different angles with the number of *M*, the receiving amplitude vector of *E_R_* (*L* × 1 dimensions) can be represented as

(2) $$E_{R}=GS+N$$

where the column vector *S* (*M* × 1 dimensions) represents the signal of the source coming from different incident angles and vector *N* (*L* × 1 dimensions) represents the noise of the environment in the angle domain in the measurement. It was worth noting that when *G* was a non‐singular matrix, the column vector *S* could be perfectly solved. However, considering that *G* does not process an inverse directly, the compressive sensing method could be applied to reconstruct the vector of *S*.

By introducing a penalty term *λ* = 0.1 and applying the Tikhonov regularization method,^[^ ^60^ ^]^ the result of vector *S* reconstruction can be given by

(3) $$S={\left(G^{H}G+\lambda I\right)}^{-1}G^{H}E_{R}$$

where the symbol *H* represents the operation of conjugate transpose and *I* is the identity matrix with the dimensions of *M* × *M*.

### The Theory of Controlling the Scattering Wave

According to the synthesized method of scattering or radiated wave in the far field, the issue of retro‐reflection could be achieved by distributing the required reflective phases of unit cells in terms of the equation given by

(4) $$\varphi_{n}=2\times k_{0}\left[\left(n-1\right)d+\frac{d}{2}-\frac{Nd}{2}\right]\sin\phi_{0}$$

where *k*_0_ represents the wave number in space, ϕ_0_ denotes the incident wave angles (the inverse angle of the retro‐reflection) in the azimuth planes, n represents the column index of the metasurface which includes *N* = 16 columns total.

The issue of low scattering could be achieved based on the concept of reflective phase cancellation of metasurface. Accordingly, the reflective phase distribution can be summarized as

(5) $$\varphi_{n}=2\times k_{0}\left[\left(n-1\right)d+\frac{d}{2}-\frac{Nd}{2}\right]\sin\phi_{0}+\left(n-1\right)\pi +\delta_{n}$$

where *n* is the column index of the metasurface, *d* is the interval between adjacent columns, and δ(*n*) represents the perturbation of the phase in the *n*th column in the measurement. Based on the phase distribution for achieving retro‐reflection mentioned above, the phase difference of πwith the perturbation of δ was introduced between adjacent columns to make the spatial cancellation for the reflection wave of the whole metasurface. For the whole metasurface, the electrical field *E_s_* of the scattering wave can be calculated according to the far‐field synthesizing method as below:

(6) $$\begin{array}{c} E_{s}\left(\phi \right)=\sum_{n=0}^{N}E_{in}^{n}\Gamma_{n}e^{-jk_{0}d\sin\phi }=\sum_{n=0}^{N}\left(e^{jk_{0}d\sin\phi_{0}}A_{n}e^{-jk_{0}d\sin\varphi_{n}}\right)e^{-jk_{0}d\sin\phi } \end{array}$$

where $E_{in}^{n}$ is the incident field and Γ_*n*_ is the reflection coefficient (with the reflection amplitude of *A_n_* and reflection phase of φ_*n*_) for each unit cell respectively.

## Conflict of Interest

The authors declare no conflict of interest.

## Supporting information

Supporting Information

## Data Availability

The data that support the findings of this study are available from the corresponding author upon reasonable request.
