# Supplementary material for: Incident Angle Sensing and Adaptive Control of Scattering by Intelligent Metasurface
Source: Adv Sci (Weinh). 2024 Aug 29;11(40):2406841. doi: 10.1002/advs.202406841 (PMC11516094; doi:10.1002/advs.202406841)
Supplement: Supplementary file 1 — Supporting Information [file ADVS-11-2406841-s001.pdf]

ADVANCED SCIENCE
Open Access

Supporting Information

for *Adv. Sci.*, DOI 10.1002/advs.202406841

Incident Angle Sensing and Adaptive Control of Scattering by Intelligent Metasurface

*Yong Han Liu, Xu Hang Li, Ze Tong Zhang, Kai Nan Qi, Lan Lu, Shi Yu Wang and Yun Bo Li**

Supporting Information

Incident angle sensing and adaptive control of scattering by intelligent metasurface

*Yong Han Liu, Xu Hang Li, Ze Tong Zhang, Kai Nan Qi, Lan Lu, Shi Yu Wang, and Yun Bo Li**

This supporting information includes:

- Supporting Information 1. Additional illustrations for the unit cell of intelligent metasurface
- Supporting Information 2. Measurement of the unit cell in waveguide
- Supporting Information 3. The experimental calibration of the whole metasurface
- Supporting Information 4. The illustration about the backside of fabricated metasurface
- Supporting Information 5. Measurement results of the random receiving patterns by metasurface
- Supporting Information 6. The sensing results of DoA by intelligent metasurface
- Supporting Information 7. Implementation of the intelligent module for sensing and feedback control

Supporting Information 1. Additional illustrations for the unit cell of intelligent metasurface

The active design on the bottom layer of the proposed unit cell is shown in **Figure S1**. During the operation of intelligent metasurface, the flow of RF energy within the unit is as follows: for the receiving chain depicted in **Figure S2**a, the EM energy will be guided into the power distribution network or not when the PIN diode is open or closed, and the phase shift is fixed without changing the states of programmable phase shifter. For the reflective chain shown in Figure S2b, the PIN diode is kept closed and reflective phases can be continuously manipulated by changing bias voltages loaded on the varactors of programmable phase shifter.

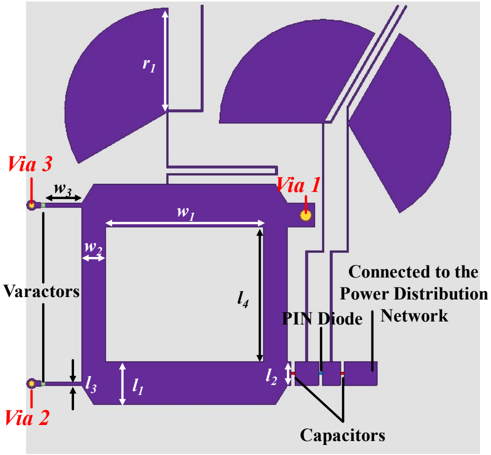

Figure S1. The bottom layer design of the unit cell integrated with programmable phase shifter and switching circuit. In which, the optimized parameters are determined as l_1=2.35 mm, l_2=1.31 mm, l_3=0.2 mm, r_1=5.6 mm, w_1=8.69 mm, w_2=1.31 mm, w_3=2 mm.

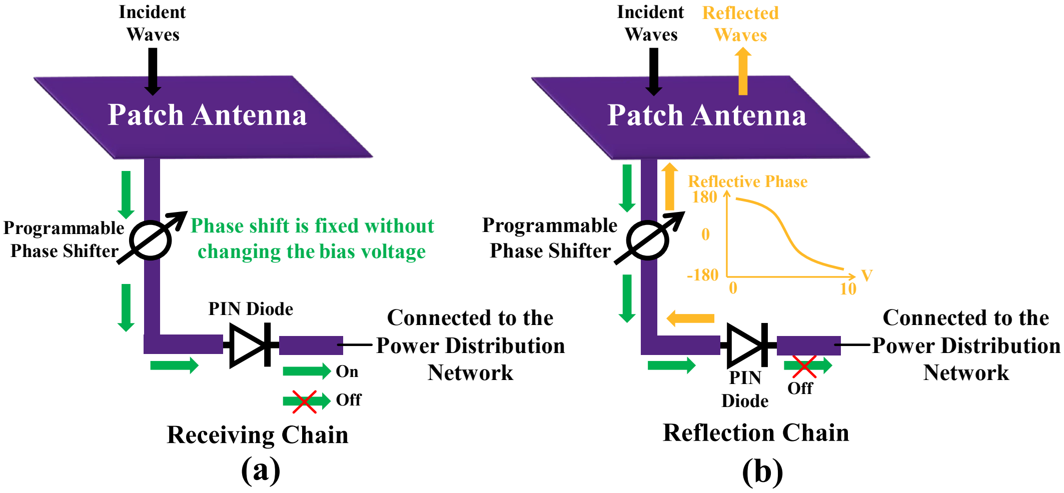

Figure S2. The schematic diagram of EM energy flow in the proposed unit cell. (a) Receiving chain; (b) Reflective chain.

Supporting information 2. Measurement of the unit cell in waveguide

A fabricated sample of the active unit cell was measured using one standard waveguide of WR-187. The experimental setup is depicted in **Figure S3**. Specifically, the metallic transition fabricated by 3D printing technology for connecting the unit cell with the waveguide is introduced. The device under test (DUT) was enveloped by metallic via holes for mimicking the boundary of perfectly electric conductor (PEC).

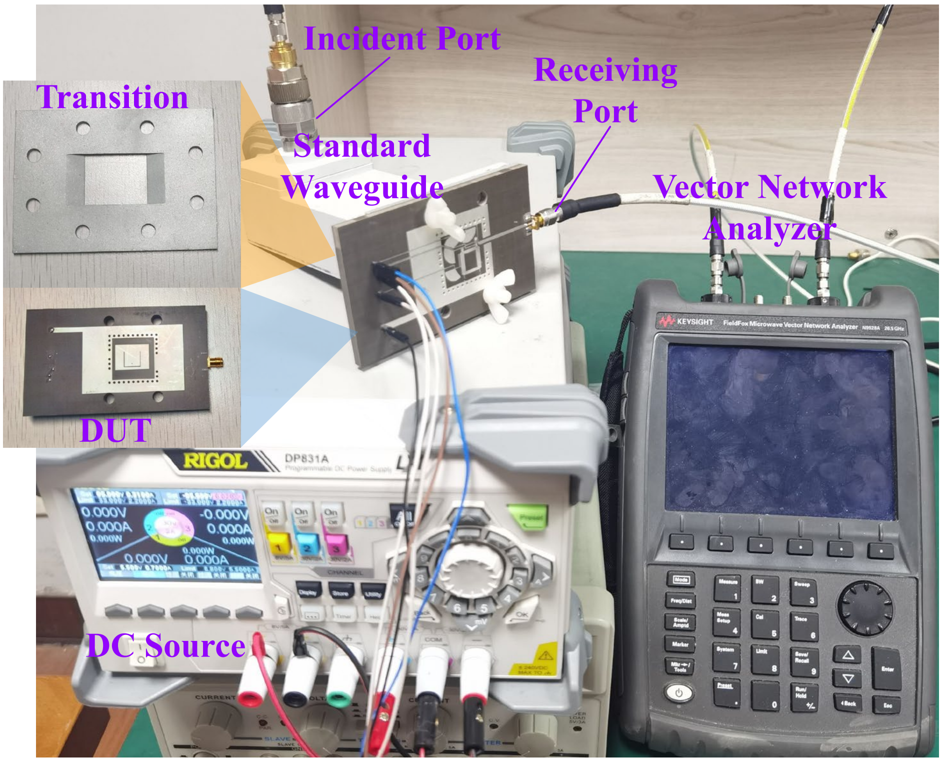

Figure S3. The experimental setup of the standard waveguide measurement.

The measurement results of reflection and receiving with changing the states of PIN diodes and varactors can be obtained by parameters of S_{11} and S_{21} respectively using vector network analyzer (VNA). The excitation of electromagnetic (EM) wave is launched through waveguide, and the measurement results of EM receiving by switching DC voltages (0 V and 1.3 V) on the PIN diode with changing the DC voltages on varactors from 0 V to 10 V, are shown in **Figure S4**a. Accordingly, the amplitude difference between the PIN states of On and Off can reach approximately 10 dB. For the measurement of EM reflection with the PIN diode voltage been kept at 0 V, the measurement results of reflective phase and amplitude with changing the DC

voltages on varactors from 0 V to 10 V are shown in Figure S4b,c. Therefore, the reflective phase can cover 360° while the amplitude fluctuation remains within 4 dB from 5.2 GHz to 5.6 GHz. For the differences between the results of waveguide measurement and simulations of the unit cell, it can mainly be attributed to the different EM boundaries of PEC and period, the different EM excitations of oblique and normal directions to the unit cell, and the errors introduced by the transition which is inevitable in our waveguide experiment.

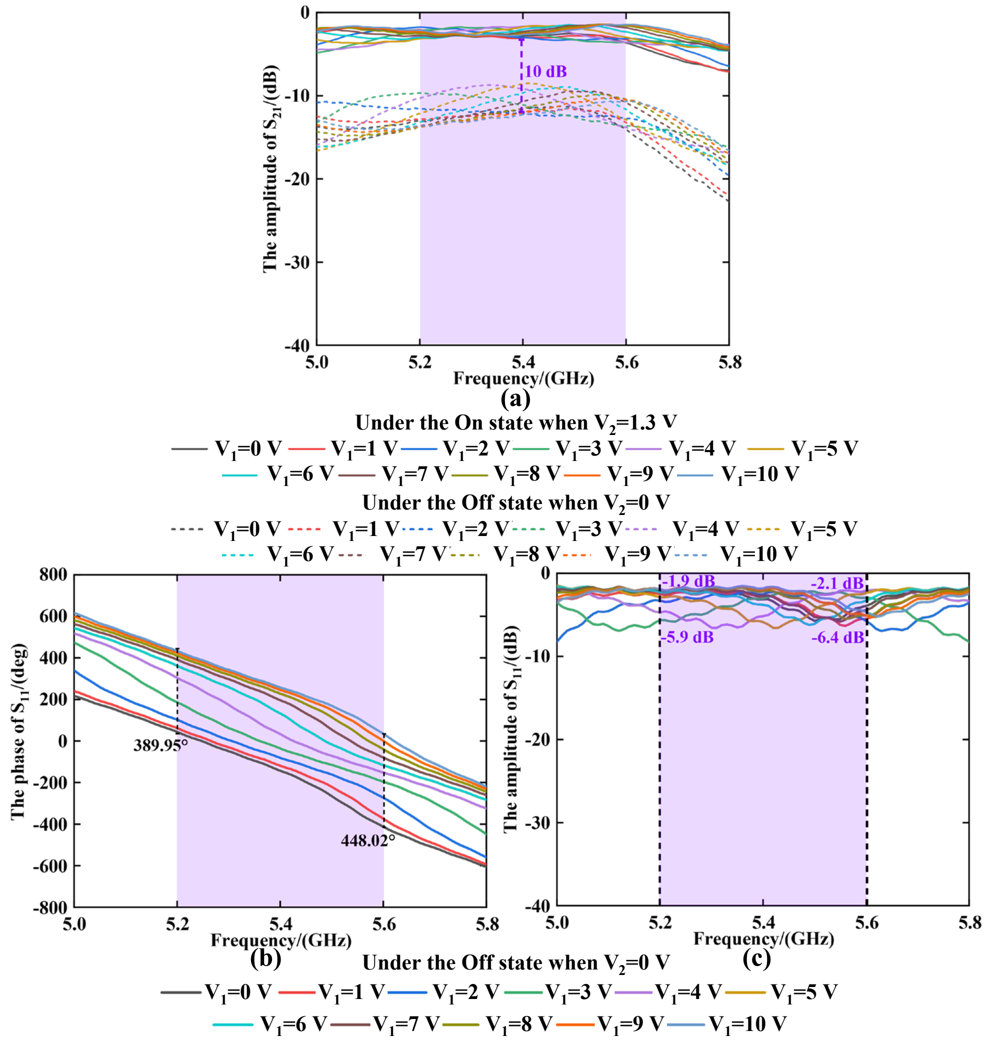

Figure S4. The measurement results of (a) the amplitude of S_{21} when the voltage of varactors (V_1) changes from 0 V to 10 V under On and Off states of the PIN diodes (V_2); (b) The reflective phase (the phase of S_{11}) and (c) the reflective amplitude (the amplitude of S_{11}) while the voltage of varactors (V_1) changing from 0 V to 10 V under the Off state of the PIN diodes.

Supporting Information 3. The experimental calibration of the whole metasurface

The illustration of the experimental calibration for the whole intelligent metasurface is shown in **Figure S5**. All of the bias lines for controlling the PIN diodes and varactors of all unit cells are connected to the same DC signals respectively.

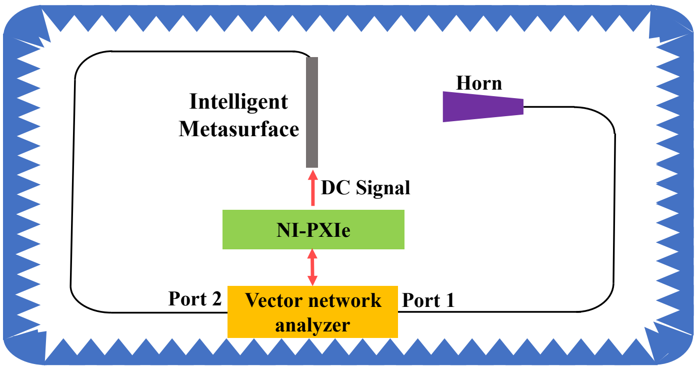

Figure S5. The schematic of the experimental calibration for the whole intelligent metasurface.

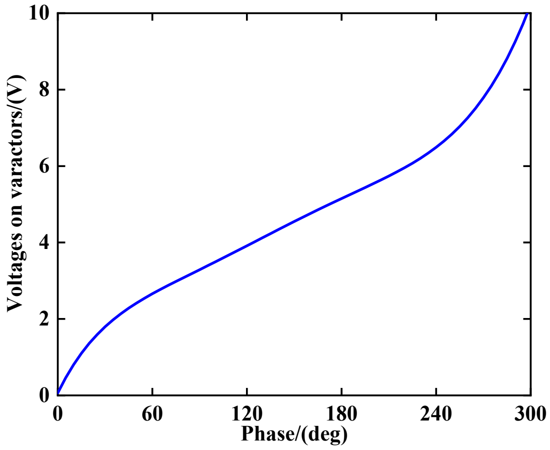

Figure S6. The curve fitting results based on calibration between the reflective phase and DC voltage under 4.9 GHz.

For the relationship between dynamic reflective phase and corresponding DC voltages shown in **Figure S6**, it can be acquired with the method of polynomial fitting based on the phase data of S_{11} collected by VNA, and the fitting result is given as

$$f(x) = p_1 \cdot x^6 + p_2 \cdot x^5 + p_3 \cdot x^4 + p_4 \cdot x^3 + p_5 \cdot x^2 + p_6 \cdot x + p_7 \quad \text{(S1)}$$

where p_1 to p_6 are the coefficients of polynomial, in which p_1 = -6.656 e-14, p_2 = 9.586 e-11, p_3 = -4.577 e-08, p_4 = 1.016 e-05, p_5 = -0.001145, p_6 = 0.08404 and p_7 = 0.06555. The variable x represents the input reflective phase and $f(x)$ is the calculated result of voltage value.

Supporting Information 4. The illustration about the backside of fabricated metasurface

The fabricated metasurface composed of 8*16 unit cells is under the column-control method, and a power distribution network of 1-to-16 combining with 16 ones of 1-to-8 shown in **Figure S7** is introduced to connect all of the unit cells together to receive the EM power into the SMA port connected with intelligent module for the sensing work. For the power distribution network of 1-to-8, it can make the 8 unit cells in column operated by uniform DC voltages on PIN diodes or varactors as one bigger unit cell. Thus, we consider the whole metasurface that is composed of 16 bigger unit cells for controlling the EM receiving and scattering. For the bigger unit cell, we only use one programmable phase shifter and switching circuit to achieve the corresponding manipulations. And the capacitors of C_1 and C_2 closed by the PIN diode in the switching circuit are applied to separate the DC supply for independent control. To prevent leakage of RF signals during loading DC voltages on the diodes, matching stubs have been strategically placed along the DC bias lines after simulating in HFSS, which act as open circuits at the junction of the bias lines and the microstrip lines.

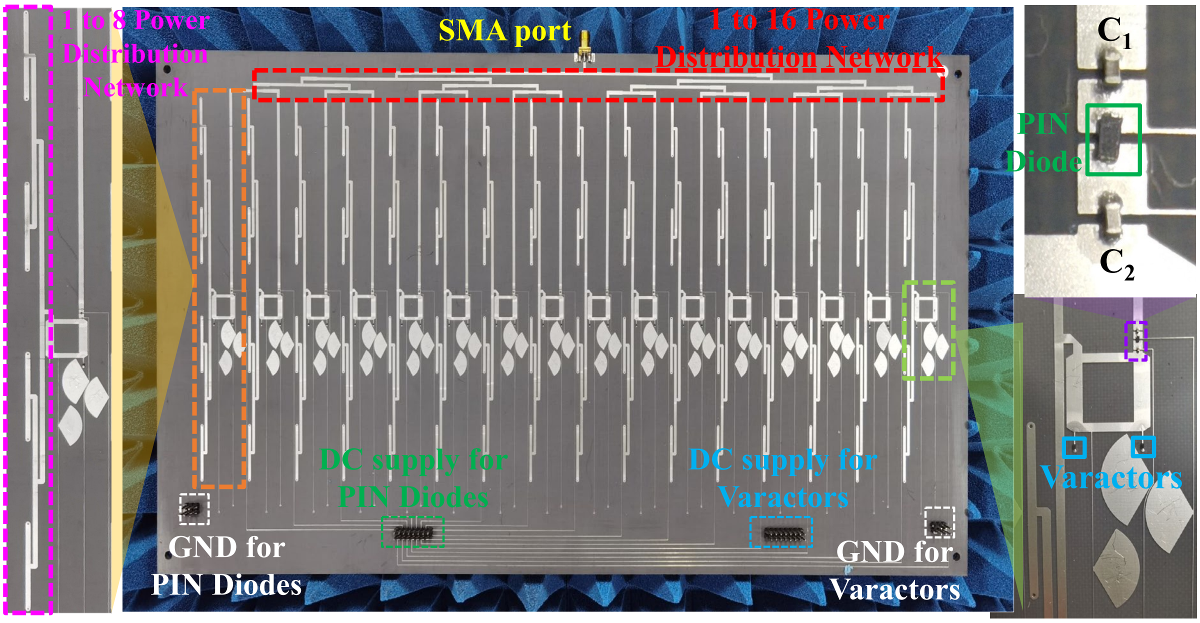

Figure S7. The photograph of the fabricated metasurface sample (bottom view) with the size of 432.7×240.8 mm^2 including biasing networks and the zoom-in view of active components including programmable phase shifter and switching circuit, in which C_1=1 nF, C_2= 1 nF.

Supporting Information 5. Measurement results of the random receiving patterns by metasurface

In our measurement, only 60 diverse receiving patterns are applied to construct the sensing matrix *G* based on the method of compressive sensing. The corresponding randomly programmable coding distributions for On and Off states of PIN diodes are stored into the register of FPGA in the intelligent module. The selected three different coded apertures (labeled as #10, #30, and #50) of column-control metasurface are shown in **Figure S8**a,b,c. Corresponding measurement results of receiving patterns are depicted in Figure S8d,e,f under frequencies of 4.8 GHz, 4.9 GHz and 5.0 GHz respectively.

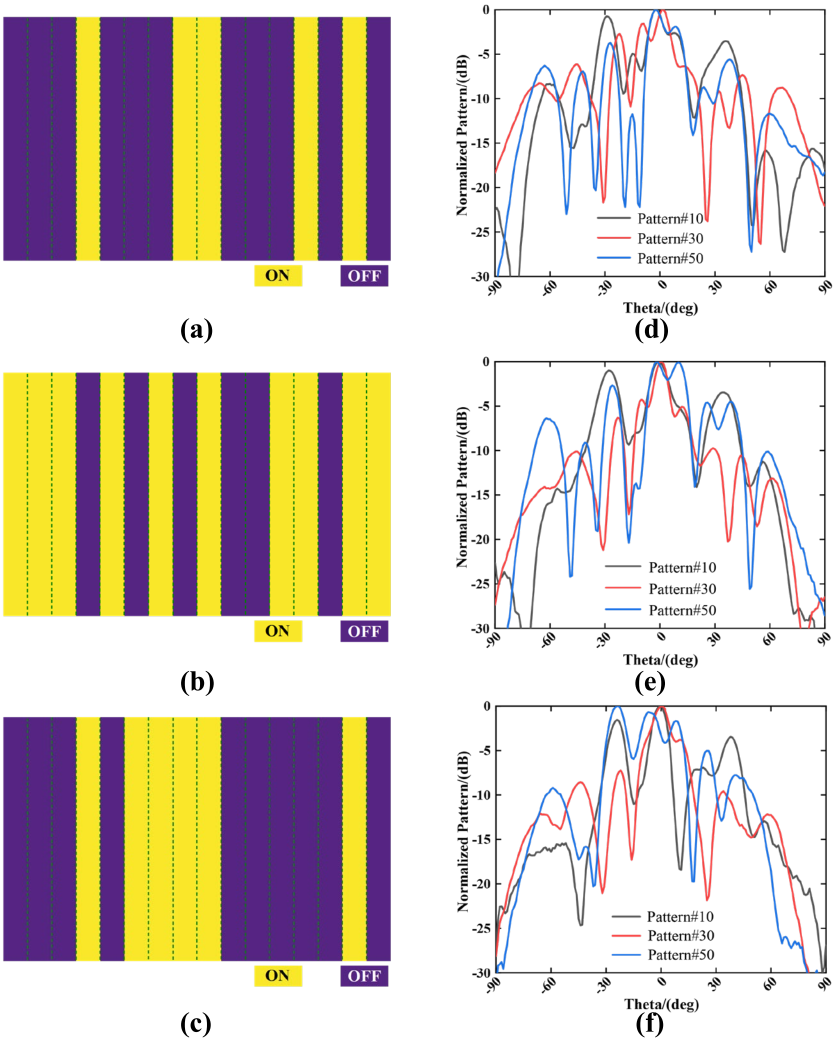

Figure S8. (a)-(c) Three selected random coding distributions of the programmable metasurface based on the On and Off states of PIN diode. The patterns are numbered as follows: pattern #10, pattern #30, and pattern #50. (d)-(f) Corresponding far-field receiving patterns under the frequency of (d) 4.8 GHz, (e) 4.9 GHz, and (f) 5.0 GHz respectively.

Supporting Information 6. The sensing results of DoA by intelligent metasurface

In the azimuth plane with the angle range from -90° to 90°, the spatial angle sensing of the source placed in the far field is accomplished by metasurface with the intelligent module located at the rotating platform. And three selected sensing results of DoA using compressive sensing method in the measurement are presented in **Figure S9**. Additionally, the sensing results of DoA and the estimation error are shown in **Figure S10**.

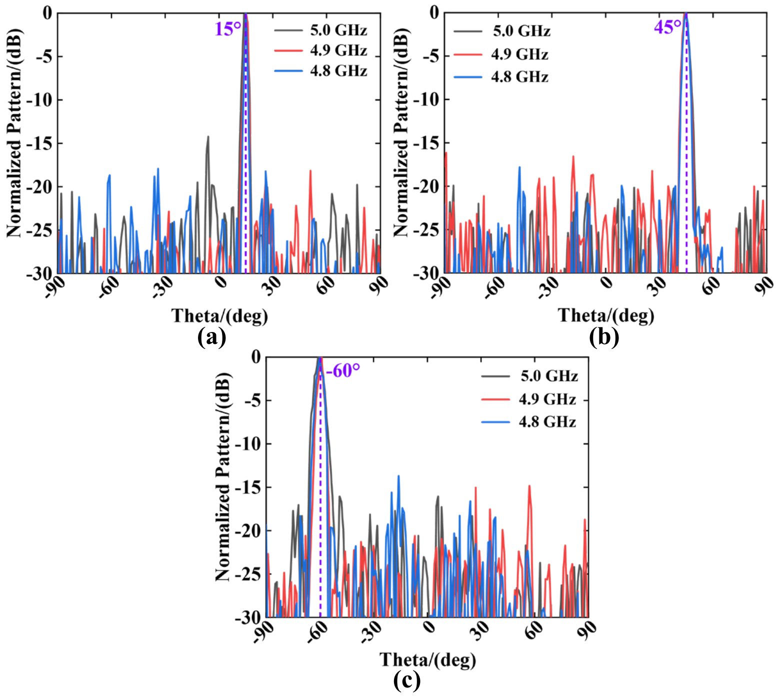

Figure S9. The sensing results of DoA based on the source located at the spatial angles of (a) 15°, (b) 45°, and (c) -60° under frequencies of 4.8 GHz, 4.9 GHz and 5.0 GHz respectively in the measurement.

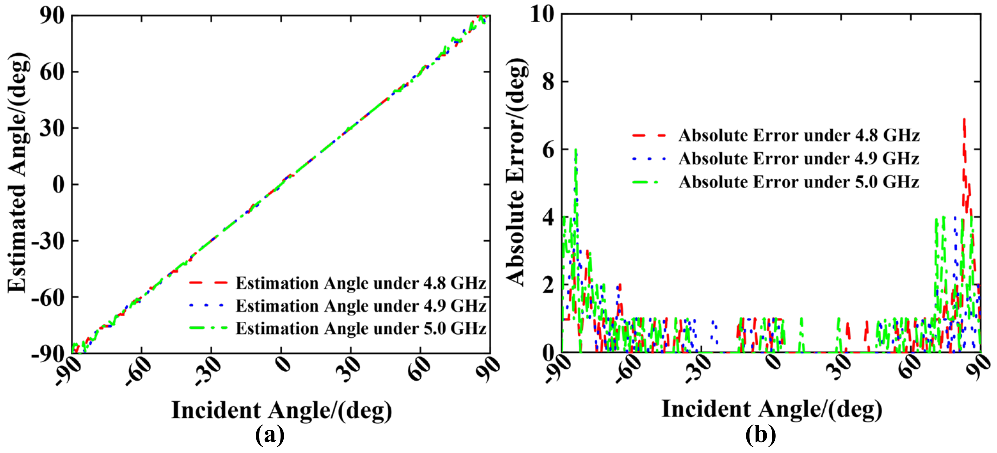

Figure S10. (a) The sensing results of DoA using compressive sensing method; (b) Absolute error of sensing results compared with the real angles.

Supporting Information 7. Implementation of the intelligent module for sensing and feedback control

The intelligent module shown in **Figure S11** located at the back of the metasurface is designed under technologies of baseband, it can realize the sensing and feedback control to make the metasurface to be intelligent.

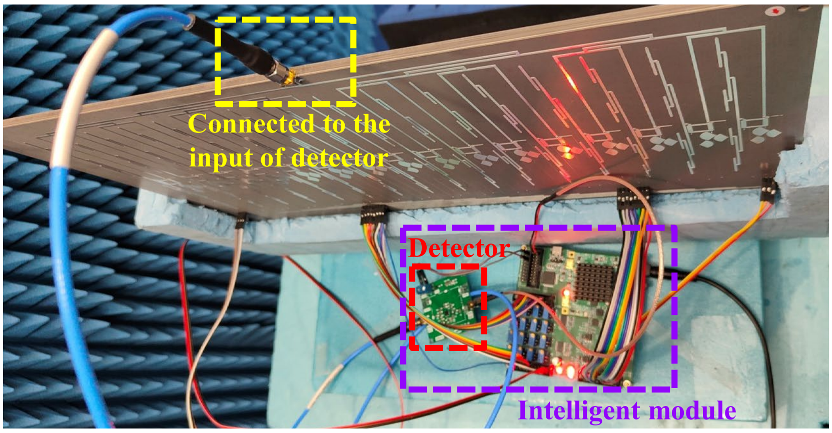

Figure S11. A photograph of the intelligent module located at the back of metasurface.

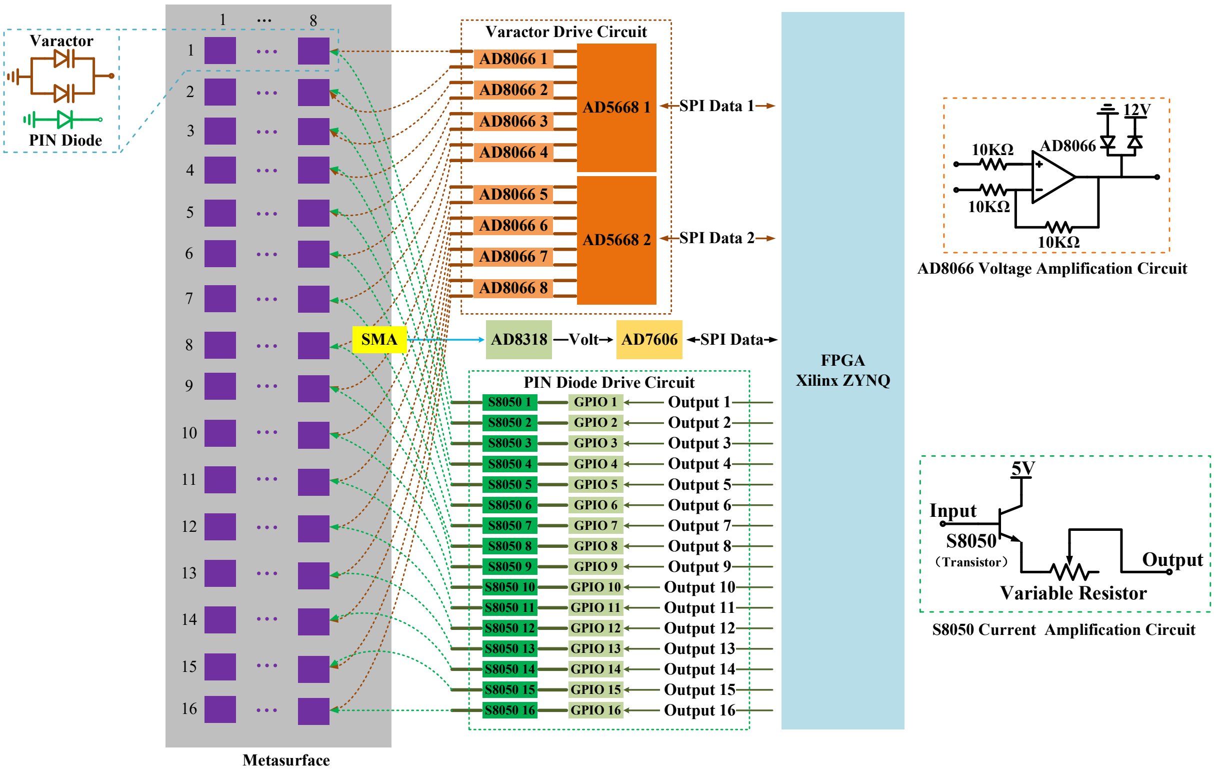

Figure S12. The schematic design frame of proposed intelligent module

The whole design frame of the intelligent module is presented in **Figure S12**. The core component is a Xilinx ZYNQ chip, which is connected to several ADC and DAC chips via SPI interface to collect and output voltages. Additionally, several transistors of S8050 are connected via GPIO to drive the PIN diodes.

The operation process of the intelligent module is as follows:

Firstly, the calibration is performed by controlling the transistors of S8050 through the 16 FPGA GPIOs to enter either amplification or cutoff states initially. For each transistor under the amplification state, it can provide 10mA current to the PIN diode. In the sensing process, the states of the 16 GPIOs are randomized to achieve random variation of receiving aperture. When the incident waves impinge on the programmable metasurface, the RF signals can be converted into DC voltage by the detector AD8318.

Subsequently, the voltage is converted into digital code by the analog-to-digital converter chip AD7606 and sent to the FPGA for storage. The entire calibration process needs to be performed at each of the 181 angles (including the angle range from -90° to 90° with the interval of 1°) for 60 times, aiming to obtain the energy receiving of EM waves by the metasurface under 60 diverse receiving apertures. Ultimately, a matrix of size 181×60, denoted as the sensing matrix G will be obtained.

Then, for the practical application, 60 GPIO are changed to generate 60 different receiving patterns and synchronous sampling of voltage using AD7606 are performed, resulting in a receiving amplitude vector E_R with 60 elements, which is then multiplied by the coefficient matrix ($(G^H G + \lambda I)^{-1} G^H$ in Equation (3) of main manuscript) to obtain the vector S

Finally, the voltages are fed back based on the information of sensing angle which are pre-stored in registers within the FPGA to load on the varactors in 16 column-control programmable phase shifters for each angle.

The internal bus architecture of the entire system is depicted in **Figure S13**, where the ZYNQ connects to 7 AXI-Lite buses to achieve data transmission and system control. 60 times of sensing operation take about 384 μs in total ($60\times(T_{GPIO}+T_{AD7606}) = 60\times(1.2\ \mu s + 5.2\ \mu s) = 384\ \mu s$), while the time required for calculation and feedback the voltage is about 1.07 ms and 5 μs respectively. Thus, the total time cost for sensing and feedback control by our intelligent module is approximately 1.459 ms.

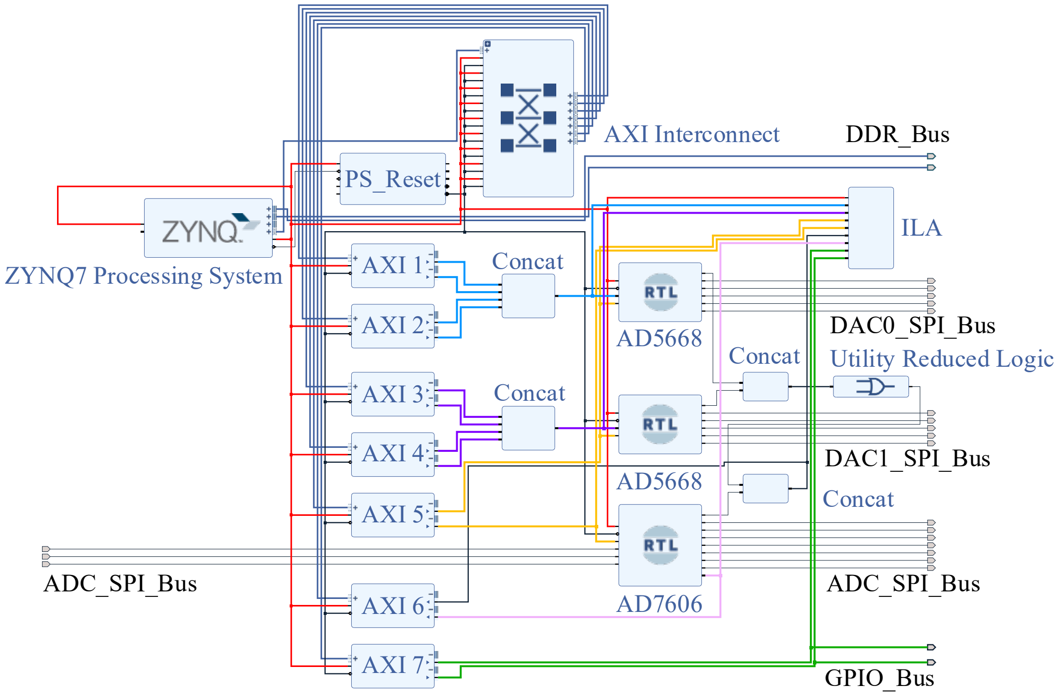

Figure S13. The architecture of internal bus for the entire system of intelligent module.

The model diagram of the intelligent module is depicted in **Figure S14**a,b. Region 1 can take the output from detectors as its input and transmits the collected voltages to the FPGA via the SPI interface. Region 2 can provide the current driving for PIN diode. Regions 3 and 6 provide the voltage driving for varactors. Region 4 is the high-speed interface connecting the ZYNQ System-on-Module (SoM), enabling the SoM to establish a connection with the intelligent sensing and feedback system through high-speed interfaces for control and data exchange. The Region 5 serves as the power network to provide the electrical power to the entire intelligent system.

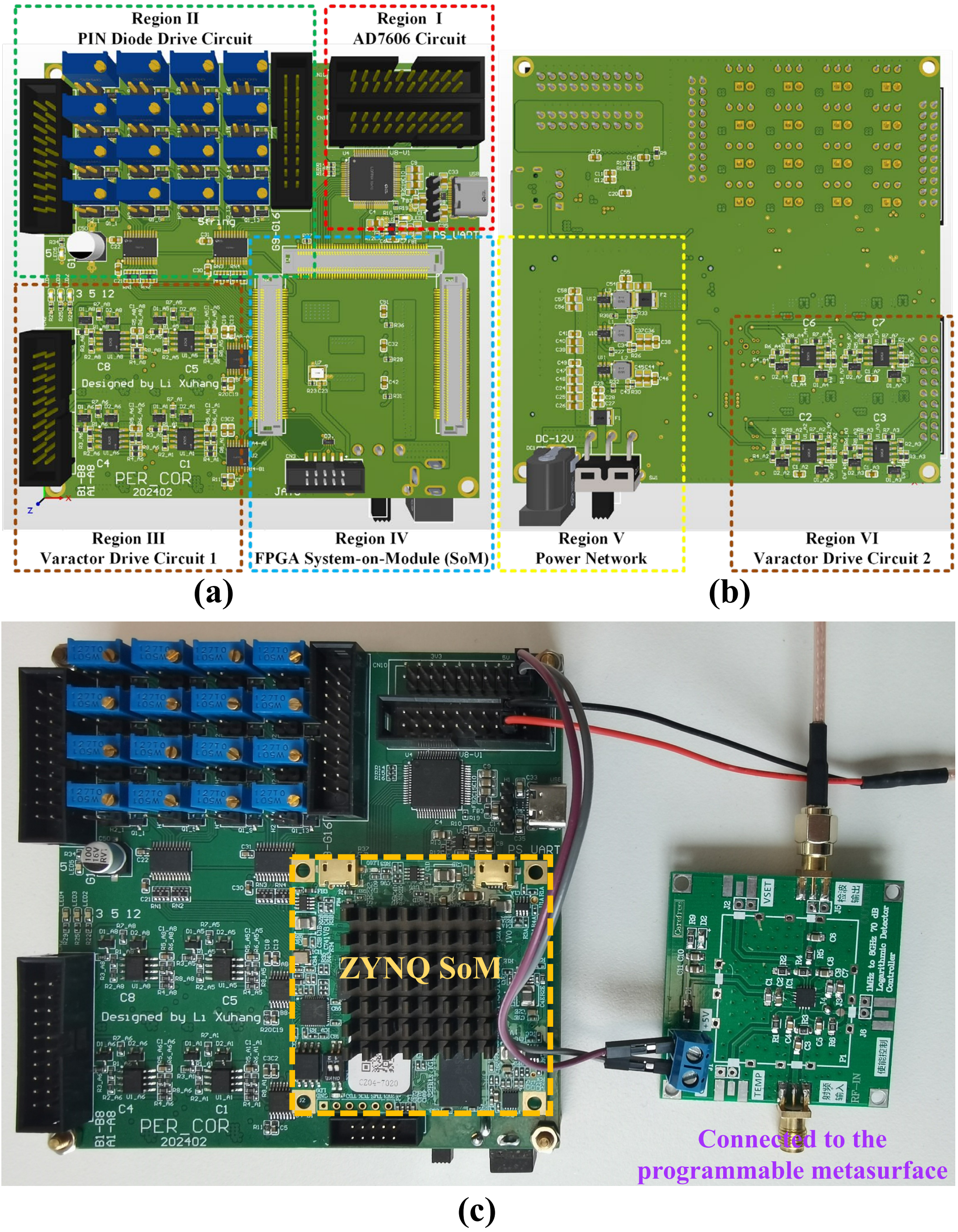

Figure S14. (a) The top view of the model design. (b) The bottom view of the model design. (c) The fabricated prototype of intelligent module.
